# Performance of innovative nanomaterials for bone remains consolidation and effect on ^14^C dating and on palaeogenetic analysis

**DOI:** 10.1038/s41598-022-10798-5

**Published:** 2022-04-28

**Authors:** Francesca Porpora, Valentina Zaro, Lucia Liccioli, Alessandra Modi, Arianna Meoli, Giulia Marradi, Serena Barone, Stefania Vai, Luigi Dei, David Caramelli, Mariaelena Fedi, Martina Lari, Emiliano Carretti

**Affiliations:** 1 Department of Chemistry Ugo Schiff” and CSGI Consortium, University of Florence, via della Lastruccia 3-13, 50019 Sesto Fiorentino, FI Italy; 2 Department of Biology, University of Florence, via del Proconsolo 12, 50122 Florence, FI Italy; 3 Q30265285 INFN (Istituto Nazionale Di Fisica Nucleare) Sezione Di Firenze, via G. Sansone 1, 50019 Sesto Fiorentino, FI Italy

**Keywords:** Genetics, Chemistry

## Abstract

An innovative protocol for the consolidation of ancient bone remains based on the use of nanometric HydroxyAPatite (HAP) was set up and tested through a multidisciplinary approach. A new protocol for the synthesis of HAP nanoparticles was developed, and the composition of the obtained nanomaterial was investigated through Fourier Transform Infrared Spectroscopy (FTIR) and X-Ray Diffraction (XRD); sizes, shape and morphology of the synthesized particles were studied by Scanning Electron Microscopy (SEM). The consolidation performance was evaluated by testing the new nanomaterial on degraded ancient bone findings. An increase of the mineral density and of the micro-hardness of the bone were observed. The new consolidation method was also tested to assess possible effects on the palaeogenetic analysis and radiocarbon dating on the treated bones. The consolidation treatment does not introduce any contaminations that could affect radiocarbon dating and has no general detrimental impact on the genetic characterization of the skeletal remains. This consolidation procedure represents a more compatible conservation tool with respect to traditional procedures: it has been shown that the treatment is effective, easily-applicable and compatible with post-consolidation analysis.

## Introduction

In the field of cultural heritage conservation, bone remains from historical, archaeological and paleontological contexts are peculiar and precious finds. Particularly, human skeletal remains represent an enormous source of information about ancient humans in terms of their evolutionary and adaptation mechanisms, migratory flows and lifestyle (social and cultural behaviours, diet and diseases)^1–7^. Similarly, animal skeletal remains can provide information on past environments and on the social and economic organization of the populations which they are associated with^8–11^.

The chemical composition of bones is characterized by packed collagen fibres interconnected with a network of microcrystals of apatite with general formula Ca_10_(PO_4_)X_2_, where X usually indicates a hydroxyl group (HydroxyAPatite, HAP). While the stoichiometric hydroxyapatite shows a Ca:P ratio of 1.67, the value typically observed in the organisms is widely variable due to several ion substitutions present in the hydroxyapatite of biological origin^12^.

Ancient bone remains are typically discovered in critical conservation conditions, mostly in direct contact with the soil. The physico-chemical characteristics of the external environment (pH of the soil, moisture, temperature, environmental redox potential), the prolonged interaction with micro-and macro-organisms, together with the intrinsic physico-chemical properties of the remains, such as porosity and crystallinity, can strongly affect the preservation of the bones^13–15^. Consequently, in view of future studies, analyses and exhibitions, the recovery and handling of these objects are often critical.

Since the beginning of the twentieth century, the products typically used for consolidation treatments and fixing of the fragments are realized by natural and synthetic organic polymers (i.e., vinyl and acrylic polymers and copolymers)^16^. Unfortunately, their scarce physico-chemical compatibility with the substrate and their low chemical stability, can lead to a rapid decrease of their performance, cause alterations of the bone matrix (yellowing and strong change of the porosity) and induce mechanical stresses due to the shrinking of these polymeric materials upon ageing. In addition, their degradation can seriously compromise their removal and the re-treatability of the object, besides hampering the analysis of biomolecules such as DNA and protein^16,17^. Moreover, the application of organic polymers may significantly alter ^14^C radiocarbon dating analysis by introducing external carbon atoms, especially in the case of poor information about the previous treatments or severe degradation processes occurred to those polymers.

In the last few years, inorganic nanomaterials have been proposed as a useful alternative to organic polymers^18,19^ for the conservation of different classes of works of art (i.e., consolidation of wall paintings and carbonate stones, and deacidification of cellulosic materials such as canvas and papers). These materials have the great advantage of physicochemical compatibility with the materials constituting the works. As an example, Ca(OH)_2_ nanoparticles dispersed in 2-propanol have been used for the consolidation of archaeological bones to promote, through carbonation induced by the atmospheric CO_2_, the formation of aragonite, a metastable polymorph of CaCO_3_ characterized by strong mechanical properties^20^. Since aragonite is not the best conservation material for bones (mainly composed by HAP), the use of HAP for consolidation purposes of archaeological bones was evaluated in more recent studies. Indeed, HAP has also recently given promising results for restoring the mechanical integrity of degraded stones^21–24^. Some studies have examined the possibility to induce the precipitation in situ of HAP: the first approach considered is the reaction between a solution of a phosphate precursor, such as DiAmmonium hydrogen Phosphate (DAP), with the calcium present in the bone^25^. Unfortunately, the magnesium cation strongly affects the HAP crystallization processes^26^, and, as in the case of many other ions that are naturally present inside the bone matrix, can influence/inhibit the formation of a crystalline network of HAP.

In recent studies^27,28^, the possibility to induce the *in-situ* growth of HAP was evaluated by immersing the bone fragments in an aqueous solution of DAP and also adding a dispersion of Ca(OH)_2_ nanoparticles in alcohol. Despite the promising results obtained in a recent study^28^, three main aspects remained to be deeper investigated and possibly solved in the sense of improving performance: (i) no reliable data were achieved about the amount of neo-formed HAP and its possible relationship with the increase of sample compactness (as deduced from BET specific area, porosity, and micro-hardness), (ii) the negative side effect of pre-treating the bone samples by Ca(OH)_2_ nanoparticles before DAP solution application, resulting in both reduced mechanical performance and structural compactness with respect to the treatment by only DAP, probably due to the occlusion of surface pores by the Ca(OH)_2_ pre-treatment inhibiting the HAP successive formation inside the bulk bone samples, (iii) whereas palaeogenetic analysis was not compromised by this new treatment, no information about ^14^C radiocarbon analysis, that is another technique very sensitive to organic treatments, were known, and (iv) the development of a new application method to treat huge and extremely fragile samples, in order to avoid consolidation by soaking. The present paper aims to study these three aspects via a novel approach that follows three innovative research strategies for the four above mentioned points: (i) separately preparing HAP nanoparticles with smaller size than Ca(OH)_2_ and applying directly them on the bone samples as the first step of the procedure, (ii) applying the Ca(OH)_2_ as second step after the HAP nanoparticles, so that inner pores were already filled by the HAP particles before the DAP solution application that was selected as the last step, (iii) adding to palaeogenetic analysis also the ^14^C dating that is well known to be extremely sensitive to organic – traditional – consolidation treatments, and (iv) applying the consolidant system only by brushes to facilitate the treatment of bones even directly on the archeological site. Therefore, the approach was truly multi-disciplinary aimed to evaluate simultaneously the improvement of the nanotechnological approach with respect to the previous work^28^, and to test the null impact on successive ^14^C dating and palaeogenetic analysis”.

The efficacy of the consolidation was examined in terms of the impact of the treatment on the physico-chemical and mechanical properties of the treated bones. Their morphology – homogeneity and surface cohesion -, porosity and micro-hardness were assessed. To investigate the impact of the consolidation treatment on the retrieval of endogenous DNA, we applied biomolecular technologies typically used in the field of ancient DNA (aDNA) to recover the mitochondrial genome (mtDNA) from both treated and untreated fragments deriving from the same bone sample. The genetic results were then compared to spot any significant impact on the quality and reliability of the genetic data. As far as radiocarbon dating is concerned, once pre-screened that the bone samples contain collagene, tests were carried out to determine whether the consolidation treatment might introduce contamination that could not be eliminated by applying the typical procedures used to extract collagen and lately purify it from natural exogenous substances^29^: to this purpose, both untreated and treated bone samples were dated.

## Materials and methods

### Chemicals

Calcium nitrate (Ca(NO_3_)_2_∙4H_2_O, 99%, Sigma Aldrich), diammonium hydrogen phosphate (NH_4_)_2_HPO_4_, DAP, 98%, Sigma Aldrich), 2-propanol (99.9%, Carlo Erba Reagents) and ethanol (99.2%, Carlo Erba Reagents) were used as received. As HAP standard we use hydroxyapatite nanoparticles purchased from Sigma Aldrich. Highly pure water (having a resistivity of 18 MΩ^.^cm) produced by a Millipore Milli-Q UV system was used during all the experiments.

### Skeletal Materials

The consolidation tests were performed on a set of human long bone fragments from two different archaeological sites (Table SI1). Fragments of the same size (of about 4 ×  2 ×  0.5 cm) were obtained from the diaphysis of each bone. The evaluation of the physico-chemical and mechanical properties, and the palaeogenetic analysis were performed on a set of femurs from Muŝov, a Longobard necropolis located in the Czech Republic. From a first macroscopic observation, the bones appeared fragmented and prone to partially break apart when handled. The impact of the treatment on mineral density, porosity and micro-hardness was evaluated on sample Muŝov66, while the palaeogenetic analysis was carried out on four different samples (Table SI1).

The radiocarbon dating was performed on three femurs from Muŝov and an additional humerus from *Porticus Octaviae*, an archaeological site in Rome (Italy) used as a common burial during the Middle Ages (Table SI1). In particular, the bone from *Porticus Octaviae* was previously restored using Paraloid B72 as a glue to stick fragments together; the restoration of the skeletal materials from *Porticus Octaviae* was conducted between 2001–2015. This consolidant is well known to be a source of exogenous carbon in radiocarbon dating when the applied collagen extraction procedure just takes possible natural contaminations into account^30^. In such a situation, to compare the impact of both Paraloid treatment and the consolidation protocol proposed in this study on radiocarbon dating, the analysis was also performed collecting a sample from the restored area.

### Synthesis of HAP nanoparticles

The nanoparticles of hydroxyapatite were synthesised by chemical precipitation from two aqueous solutions of calcium nitrate [(Ca(NO_3_)_2_∙4H_2_O)] and diammonium hydrogen phosphate [(NH_4_)_2_HPO_4_, DAP], 1 M and 0,6 M, respectively^31^. The concentration was chosen to obtain a Ca:P ratio equal to 1.67^32,33^, which is the atomic ratio of stoichiometric hydroxyapatite. For the synthesis of nanometric HAP, the DAP solution was put in an ultrasonic bath (Elmasonic S 30H, *Elma*) with a power of 80 W^34^. Thereafter, the same volume of the Ca(NO_3_)_2_∙4H_2_O solution was added in a single quick step. The fast addition of the second precursor is fundamental to induce the formation of a huge number of nuclei at the same time and favour the production of a population of small particles with low polydispersity^18^. To study the effects of different experimental conditions on the resulting particles, several syntheses were performed changing step by step a single parameter while maintaining constant the others. The investigated parameters were the temperature (25 and 50 °C; the temperature was maintained constant by using a thermostatic bath), the pH (between 9 and 11), which was adjusted by dripping ammonium hydroxide 2 M in each precursor solution, and the ageing time (between 60 and 30 min). Finally, to avoid any interference of multi-ion doping during the HAP crystals formation, no ions different from that needed for making the HAP synthesis to occur were present. The experimental conditions of each synthesis are resumed in Table 1.

**Table 1:** Experimental conditions for the synthesis by chemical precipitation from two aqueous solutions of Ca(NO_3_)_2_∙4H_2_O and (NH_4_)_2_HPO_4_.

Synthesis no	Temperature (°C)	pH	Ageing time (min)
1	25	9	60
2	50	9	60
3	25	11	60
4	25	11	30

After the reaction, the precipitate was washed six times with ethanol and five times by water and, after each washing, it was separated by centrifugation at 5000 rpm for 5 min using a Centrifugette 4206 (*Thermo Electron Corporation, ALC*). Finally, the particles were dried overnight in an oven at 80 °C. The particles were applied as dispersions in 2-propanol (concentration 1 g/L). Before the application, the dispersions were sonicated in a digital sonifier (S-250D, *Branson*) with a power between 90 and 100 W (that corresponds to an amplitude value equal to 20%) for 1 min.

### Bones consolidation

First, the surface of the bone fragments was cleaned by brush to remove dust and soil residues. For each bone, two samples of similar dimensions (of about 4 × 2x0.5 cm) were cut from the diaphysis using diamond wheels mounted on a dental device (Marathon-Multi 600 Micromotor): one piece was left untreated (NT samples) and the other one was consolidated (T samples)^28^, through consequential application by brush of the three following systems:

- a 1 g/L dispersion of HAP nanoparticles in 2-propanol (prior to apply the nanoparticles, the dispersion is sonicated again to flake off possible aggregates^35^);
- a 0.05 g/L dispersion of Ca(OH)_2_ nanoparticles in 2-propanol;
- a 1 M deionized water solution of DAP.

The three above mentioned systems were applied by 10 (HAP), 2 (Ca(OH)_2_) and 10 (DAP) brushstrokes. The number of treatments corresponded to the maximum amount of brushstrokes that it was possible to apply without having perceptible color changes to human eye (ΔE < 2); moreover, in order to maximize the physico-chemical compatibility of the consolidation treatment, the amount of applied Ca(OH)_2_ nanoparticles was minimized. Each application was carried out only after the complete evaporation of the previously applied solvent from the bone fragment. After that, samples were maintained at room temperature for one week and then kept for two weeks in a dryer at RH = 75% before evaluation.

### Analytical techniques

#### Fourier Transform Infrared Spectroscopy Measurements

Fourier Transform Infrared Spectroscopy (FTIR) spectra were collected to compare the molecular composition of products obtained by the various synthesis protocols. FTIR measurements were performed using a BioRad FTS-40 spectrometer in the range 4000–400 cm^-1^. Spectra were averages of 64 scans recorded in absorbance mode with 2 cm^-1^ resolution. KBr pellets were prepared by finely grinding and mixing few milligrams of bone powder (1–5 mg) and 200 mg of pure KBr.

#### X-ray diffraction

Powder X-ray diffraction (XRD) analyses were carried out to define the mineralogical phases obtained by the synthesis. Few milligrams of bone powder were finely ground in a mortar and analysed at the CRIST Centre of the University of Florence (Italy). A Bruker D8 Advance diffractometer equipped with a Cu Kα radiation, and a Lynx Eye detector was used operating in θ-2θ Bragg–Brentano geometry at 40 kV and 40 mA, in the range of 10–60° with a step size of 0.035° and a count time of 0.3 s.

#### Scanning electron microscope

A FEG-SEM ΣIGMA (*Carl Zeiss*, Germany), equipped with a detector INCA X-act (Oxford Instruments) for EDX analysis, was used to collect micrographs of bone samples before and after the consolidation treatment using an acceleration potential of 10 kV and a working distance of 1.4 mm. In addition, dispersions 0.04 g/L of the obtained products in 2-propanol were first ultrasonicated by a digital sonifier at the amplitude of 20% (power between 90 and 100 W) for 1 min, then a droplet of each dispersion was deposited onto a stub and left to dry. Size data used for statistical analysis were extracted from SEM micrographs by using the Image J software.

#### Turbidimetry measurements

Turbidimetry measurements were performed with a UV–Vis Evolution 220 Spectrophotometer (*Thermo Scientific*) equipped with a Xenon lamp, measuring the absorbance of the sample at 640 nm as a function of time. At this wavelength, the hydroxyapatite does not show electronic transition phenomena and the absorbance is only due to turbidity phenomena. *Pseudo-*absorbance was assumed proportional to the system turbidity: the decrease of absorbance over time is due to particles sedimentation^36,37^.The stability of two dispersions of the obtained particles in water and 2-propanol (2 g/L) was evaluated. Every system was sonicated at 20% (power between 90 and 100 W) of amplitude for 1 min and the analysis was performed at room temperature for 2400 s with an interval of 10 s between each measurement.

#### X-ray microtomography

X-ray microtomography (μ-CT) measurements, that permit to evaluate the penetration capacity of the consolidant and to define the consequent variation of mineral density and porosity of the bone, were carried out with a Skyscan 1172 high-resolution MicoCT system at CRIST Centre, University of Florence (Italy) on a sample of ~ 10 × 5x5 mm. This system has an X-ray tube with a 5 μm focal spot size. The X-rays tube equipped with a tungsten anode was operated at 100 kV and 100 μA. Placing the sample between the X-ray source and the CCD detector, 2D X-ray images were captured over 180-degree rotating sample with a slice-to-slice rotation angle of 0.3. Each 2D image represents one slice and has an acquisition time of approximately 3 s. The spatial resolution of the image was kept in a range of 5 μm in terms of pixel size.

The 3D image was reconstructed from the projections using the Nrecon software (Bruker μ-CT 1.6.10.2), which allows adjusting reconstruction parameters (smoothing, beam-hardening, ring artifact, misalignment compensation). After reconstruction, the image was analysed to obtain information on the bone structure through the CTAnalyser software (Bruker μ-CT 1.18.8.0). A 3D representation in false colour was realized by the CTVox software (Bruker μ-CT 3.3.0) to graphically identify regions with different densities.

#### Gas porosimetry

Pore size distribution measurements were performed via the N_2_ adsorption method using a Beckman Coulter SA-3100 Surface Area analyser. With the 10 points fitting carried out in the linear region of the isotherm it was possible to obtain the specific surface area through Brunauer Emmet and Teller (BET) theory^38^. The pore size distribution was calculated from the desorption branches of isotherms by means of the Barret, Joyner and Halenda (BJH) method^39^. The bone samples (0.3–0.4 g of bone fragments of few millimetres) were outgassed before analysis in vacuum conditions at a temperature of 40 °C for 12 h.

#### Vickers Micro-hardness Measurements

The effect of the consolidation treatment on the mechanical properties of the bones was investigated through Vickers micro-hardness measurements. The measurements were carried out at room temperature through an HX-1000 TM (*Remet*, Italy) micro-hardness tester using a Vickers square-based diamond pyramid indenter and applying a load of 25 g for 15 s. The images were analysed using Autovickers® software. The data were obtained without any previous preparation of the sample, performing 10 measurements onto the surface, and calculating the corresponding average value and standard deviation.

#### Palaeogenetic analysis

Treated (T) and untreated (NT) bone fragments from the necropolis of Muŝov were processed at the Molecular Anthropology and Palaeogenetics Laboratory of the University of Florence to test whether the consolidation treatment could in any way affect the outcomes of the palaeogenetic analysis.

After the consolidation procedure, both T and NT samples were superficially cleaned by removing ca. 1–2 mm of bone surface using disposable rotary tools mounted on the same dental device previously used to cut the bone fragments. Decontamination of the samples was performed under ultraviolet (UV) light at 254 nm before proceeding with the collection of approximately 50 mg of bone powder from the inner part of the fragments. DNA was extracted from the bone powder following a protocol commonly used in the field of ancient DNA to optimise the retrieve of short molecules^40^; 20 µL of each extract were converted in double stranded and dual indexed genomic libraries^41^. No UDG treatment was performed to preserve nucleotide misincorporation patterns. Libraries were subsequently enriched for mitochondrial genome by applying a capture protocol developed by Maricic and colleagues^42^. Blanks have been included in the experimental steps and processed alongside the samples to trace any possible contamination occurred during the laboratory procedures.

The captured libraries were pooled in equimolar amount and sequenced in paired-end mode on an Illumina MiSeq instrument (2 × 75 + 8 + 8 cycles) for a depth of ~ 1 million reads for sample. Raw reads generated from the sequencing were processed through the EAGER software (v 1.92.55), following a pipeline specifically developed for the analysis of aDNA^43^. In particular, aDAPter sequences were removed and the merging of paired-end sequencing data with Clip&Merge was performed by retaining only read pairs with a minimum overlap of 10 bp. Moreover, DNA sequences shorter than 30 bp were discarded. After this filtering step, merged reads were mapped against the revised Cambridge Reference Sequence (rCRS [NC_012920.1]) using CircularMapper, a tool specifically designed for mapping against circular genomes. Only reads with a mapping quality > 30 were kept and clonal molecules were removed with DeDup. Afterwards, MapDamage 2.0^44^ was used to check for the presence of typical aDNA damage patterns such as short length of the reads and deamination rates increasing at the ends of the fragments^45^.

Mitochondrial DNA consensus sequence was reconstructed using samtools (v 1.7) in combination with bcftools (v 1.12)^46,47^ and applying a quality filter of at least 30 to retain only high confidence calls. The mtDNA haplogroup was assigned with Mitomaster^48^ and the mitochondrial data was authenticated by estimating the proportion of human endogenous reads using ContamMix^49^.

Furthermore, to prevent our observations from being influenced by potential bias associated with the different number of raw reads generated for each sample, the EAGER pipeline was run again after having down sampled the merged data using seqtk (https://github.com/lh3/seqtk). This analysis was carried out only on the two best preserved samples (Muŝov66 and Muŝov71) and the merged data were down sampled to the lowest number of merged reads observed among the NT and T fragments of the same bone before subsampling.

#### Radiocarbon dating

After collagen extraction, radiocarbon concentrations in the selected bone samples were measured by Accelerator Mass Spectrometry (AMS), using the dedicated beam line installed at the 3 MV Tandem accelerator in Florence^50^. Collagen was collected from bone powder according to the following procedure, which is intended just to remove contamination due to the natural environment:

- demineralisation at room temperature in 1 M HCl aqueous solution for at least 24 h;
- purification of the extracted material in 0.1 M NaOH aqueous solution for 2 h and possibly in 1 M HCl for 2 h;
- gelatinisation of the residue at pH = 3 in oven at 80 °C.

Carbon was then extracted from the recovered collagen by combustion in a CHN elemental analyser Thermo Flash EA 1112 and lately converted to graphite by reaction with H_2_ in presence of Fe as catalyst^51^.

^14^C/^12^C and ^13^C/^12^C were measured along the AMS beam line to correct for isotopic fractionation. NIST Oxalic Acid II (SRM 4990C) and IAEA C7 were used as primary and secondary standards, respectively. Blank samples, with nominally no ^14^C in, were also measured to correct for background counts.

## Results and discussion

### Characterisation of products

The first aim of this study was the synthesis of very small HAP nanoparticles, to be used as physico-chemically compatible consolidant for degraded bone remains. To maximise the penetration of the HAP nanocrystals into the porous matrix of the bones, it was mandatory to minimise the dimensions of the synthesised objects as much as possible. To optimise the synthesis procedure, the effects of the experimental conditions (such as temperature, pH and stirring procedure) on the composition and the particle size of the products were investigated.

**Figure 1:**
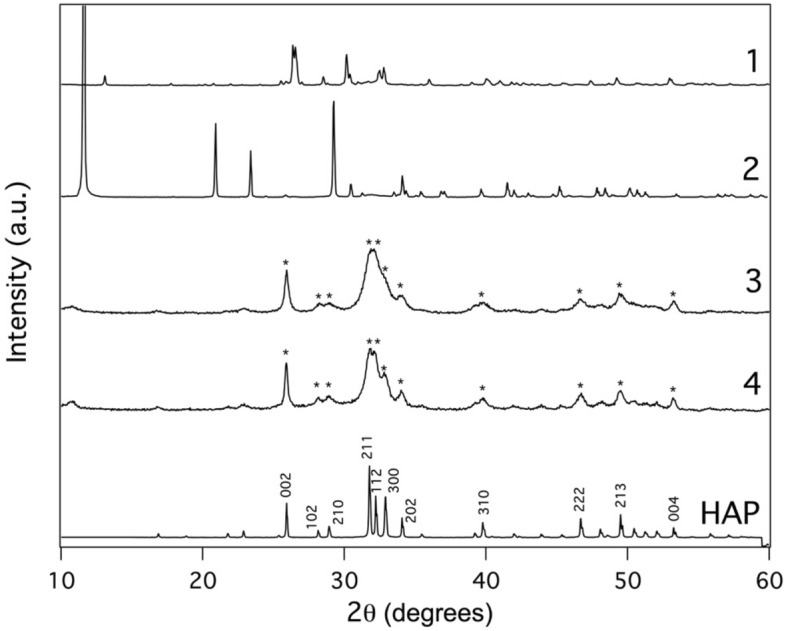
XRD patterns of the particles obtained from the syntheses 1, 2, 3 and 4 and a pattern of HAP standard purchased from Sigma Aldrich. The symbol “*” indicates the typical peaks of hydroxyapatite (JCPDS no. 9–0432).

To identify the mineral phase of the obtained products an XRD investigation was carried out (Fig. 1). In particular, the XRD patterns of syntheses 1 and 2 (that were carried out at T = 25 °C and 50 °C respectively and pH = 9 with an ageing time of 60 min, Table 1) indicated respectively the presence of monetite (CaHPO_4_, JCPDS no. 9-0080) and brushite (CaHPO_4_*2H_2_O, JCPDS no. 9-0077) as the main phases. Nevertheless, the data indicate the formation of pure hydroxyapatite from syntheses 3 and 4 (JCPDS no. 9–0432), as it was evident also by comparing the corresponding XRD patterns with the one collected from a sample of standard HAP and as confirmed by the FTIR spectra (Figure SI1 and Table SI2). This result confirmed what recently found by other Authors^52^ that showed the strong effect of pH (above 11) in determining the formation of HAP, instead of other metastable phases. This result is in contrast with what found when applying DAP on marble, probably since in our case the reaction occurred in homogeneous solution obtaining results in agreement with the data in the literature^53^, while in the case of marble the reaction proceeded by an epitaxial mechanism via solid–liquid interface and a too alkaline environment may alter the mutual stability of HAP against metastable phases^54^. According to the literature^32,34^, temperature and pH are parameters that strongly affect the mineral phase of the final synthesised product, while the ageing time does not seem to affect the mineral composition. It was interesting to notice that in this study pH = 11 at 25 °C promoted the formation of pure hydroxyapatite. On the contrary, working at pH = 9 at 50 °C and 25 °C induced the selective formation of brushite or monetite (also with the presence of some impurities) respectively, that, in this context, must be considered as undesired products. Nevertheless, these minerals are used in biomedicine for orthopedic and dental applications, such as bone regeneration, as well as for other biotechnological uses (drug delivery, cancer therapy and biosensing)^55–57^. Therefore, the development of a new procedure for the synthesis of these nanoparticles based on the improvement of the present results could be an interesting subject for further studies.

**Figure 2:**
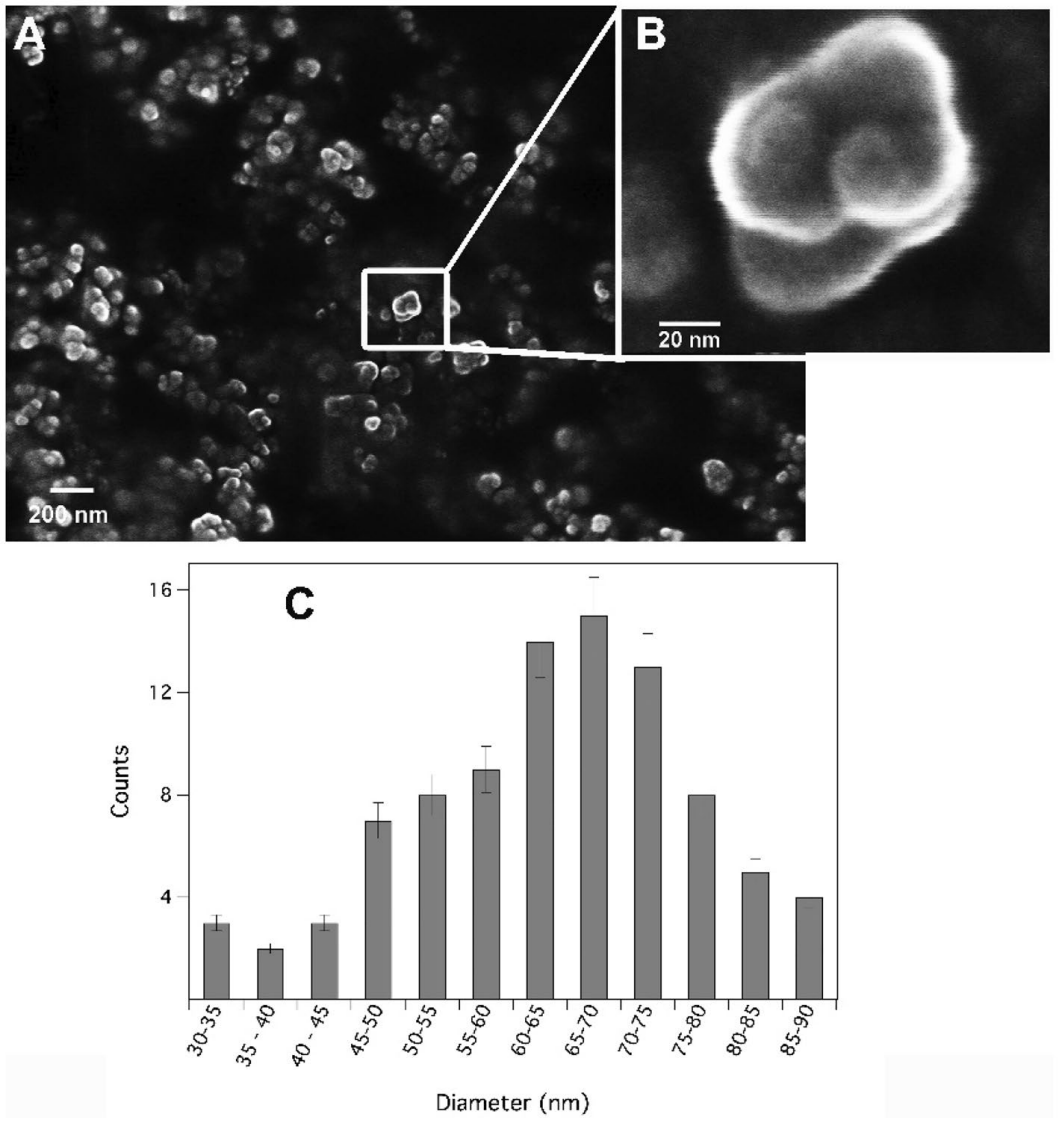
SEM micrographs of HAP nanoparticles obtained from synthesis 4 collected at different magnifications: 10^5^ X (**A**) and 10^6^ X (**B**); (**C**) size distributions of HAP nanoparticles from synthesis 4 dispersed in 2-propanol obtained by the SEM micrographs and elaborated through ImageJ.

To obtain information about the size and the shape of the synthesised HAP particles, SEM analysis was carried out on the products of syntheses 3 and 4 (Figure SI2 and Fig. 2). Both the size distribution and the polydispersity (given by the width of the distribution) did not seem to sensibly vary with the ageing time. Moreover, in both cases, HAP particles appeared as pseudo-spherical crystals with dimensions centred at 65–75 nm and ranging in the order of few tens of nanometres. EDX analysis shows that the Ca:P ratio was 1.58 ± 0.05 that, in the limit of the experimental error, is very close to 1.67 (i.e. the stoichiometric HAP Ca:P ratio) suggesting the presence of HAP, as indicated by XRD (Fig. 1). For application purposes, HAP nanoparticles obtained through synthesis 4 were used due to the shorter ageing time. Additional tests suggested that the application of ultra-sonication during the synthesis process reduced both the dimensions of the HAP nanoparticles and their aggregation, since the acoustic cavitation phenomenon, induced during sonication, promoted the formation of a high number of disaggregated HAP nanocrystals (Fig. 2A and B, Figure SI2 and SI3).

The HAP nanoparticles were applied as dispersions in 2-propanol. The stability of this system was verified through turbidimetry measurements and compared to dispersions in a different solvent like water (Figure SI4). This is a key point because the settling process is strictly related to the rate of aggregation of the nanocrystals: as faster is the sedimentation of the nanoparticles, as higher is the aggregation degree that inhibits their penetration into the porous matrix of the bone. The HAP nanoparticles dispersed in 2-propanol resulted stable up to 2500 s, while in water they sedimented after few minutes (Figure SI4).

### Evaluation of the impact of the consolidation treatment on the physico-chemical and mechanical properties of bone

**Figure 3:**
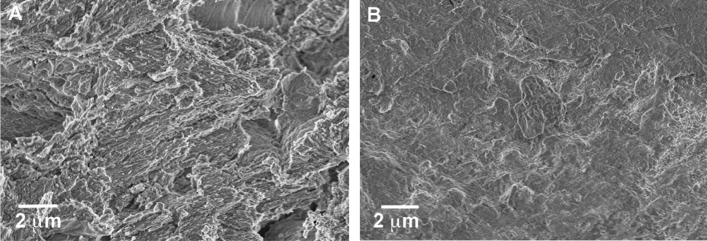
SEM micrographs registered at 10 kx of magnitude of two samples of bone from Muŝov66 before (**A**) and after (**B**) the consolidation treatment.

To evaluate the effectiveness of the consolidation protocol, bone fragments from sample Muŝov66 (Table SI1) were analysed before (NT) and after (T) the consolidation treatment by using multiple techniques that give complementary information. SEM micrographs showed that before the consolidation the sample surface appeared inhomogeneous and with many cavities and fractures (Fig. 3A), while after the treatment surface morphology was more compact, with an apparent decrease of the fraction of the open pores (Fig. 3B and SI5).

**Figure 4:**
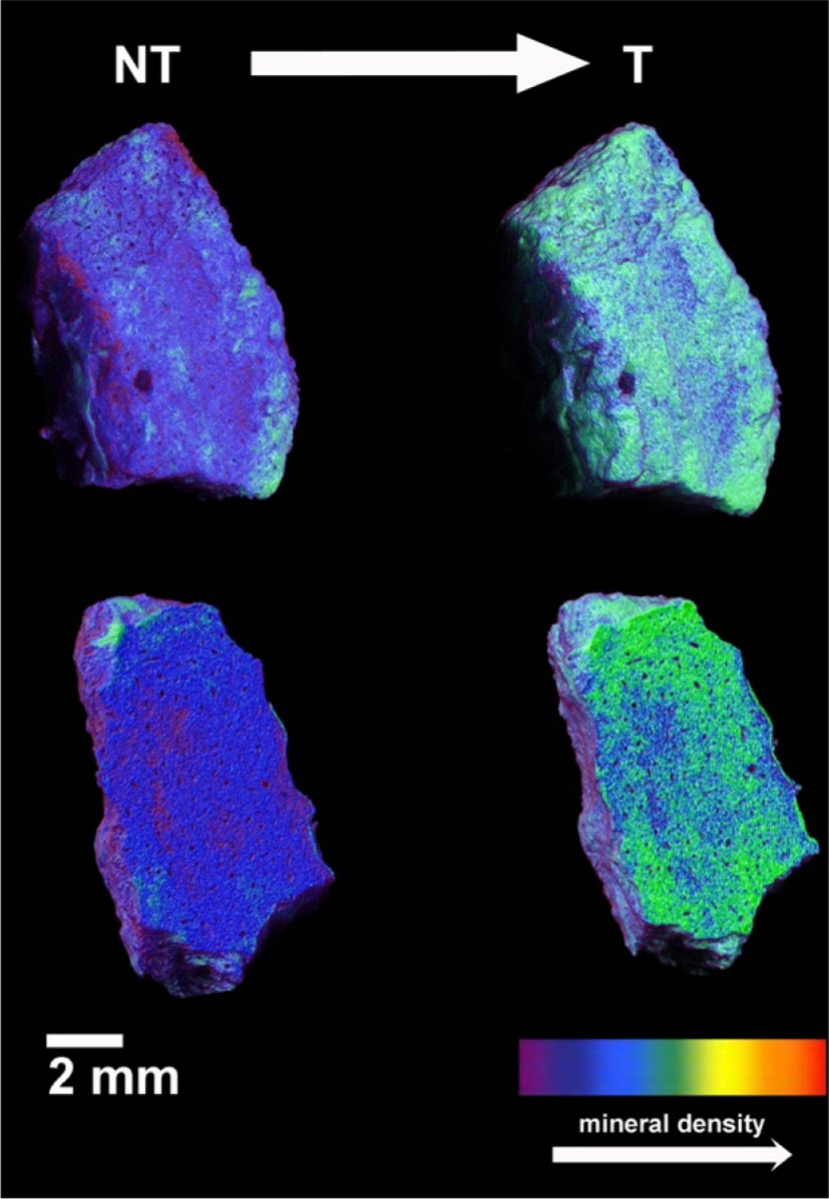
3D reconstructions in false colour of a bone fragment from the archaeological site of Muŝov (Mušov 66) before (left) and after (right) the consolidation treatment: less dense regions (in purple-blue) decrease after the consolidation in favour of a denser phase (in cyan-green). The images represent a side (top) and a section (down) of the sample.

Microtomography analysis showed a decrease of less dense regions in favour of denser ones and more compact regions in all the volume of the sample after the consolidation (Fig. 4), thus demonstrating the capacity of this treatment to deeply penetrate the bone matrix. This was also confirmed by the three sections of the 3D reconstruction of the examined bone samples before and after the treatment (Figure SI6): it was possible to appreciate an increase of the mineral density in the overall volume of the sample. Therefore, the penetration of the treatment resulted homogeneously distributed inside the bones without forming any crust at the surface layers. Consequently, the porosity decrease was homogeneously distributed over the entire sample volume, and not due to the formation of thick crusts that would be detrimental for treatment efficacy and durability. Moreover, comparing the observed porosity decrease (from 3.84 to 2.25%) with some data reported in the literature for some normal bones allows to conclude that the porosity of the treated sample remained in the range of the desired values (1.11–7.01%)^58^. These results confirmed that HAP nanoparticles followed by Ca(OH)_2_ nanoparticles and DAP aqueous solution treatment succeeded in being well integrated within the complex bone structure.

The tomography analysis also provides data about the porosity with a diameter over 5 µm (Table SI3), that is correlated to the material real density and to its compactness. After the treatment, a decrement of the total porosity (from 3.84% to 2.25%) and of the open porosity (from 3.48 to 1.31%) was measured in the overall volume of the examined sample, confirming the capacity of the treatment to deeply penetrate the bone matrix. Moreover, the observed increase of the closed porosity, from 0.38% up to 0.96%, cannot be univocally attributed to the conversion of open pores into closed pores. In fact, it was impossible to discriminate between the situation where biggest open pores are partially occluded generating new closed pores, or opened pores connected by channels below the detectability threshold. Additional information on the porosity variation detected by gas porosimetry are reported in the SI (Fig. SI7).

An increase in the average value of the Vickers micro-hardness of about 64% (from 56 ± 3 to 92 ± 3) was reported in the consolidated sample. From this standpoint, this datum indicated that the method described here represented an improvement of the procedure reported in a previous paper^28^. This result can be interpreted in terms of an increase of the mineral density and a decrease of the total porosity of the bone induced by the proposed treatment (Table SI3).

It is worthwhile to underline that this was the first consolidation method for bone remains based on the application of HAP nanocrystals followed by a mixture of Ca(OH)_2_ nanoparticles and subsequent DAP aqueous solution. Since the observed results represented truly a significant improvement with respect to the previous approach^28^ (increase of compactness as deduced from micro-hardness + 64% *versus* + 42% and + 56%, homogeneous distribution (from µ-CT) of the consolidant within the whole bone sample *versus* mainly surface consolidation, no negative effects due to pore occlusion by Ca(OH)_2_ nanoparticles), it could be interesting to further study why this sequence HAP nanoparticles application, followed by Ca(OH)_2_ treatment, and at the end by DAP solution, succeeded in giving such positive results solving the problems encountered in the previous work^28^. A hypothesis that could be the starting point for a new study could be the following. The HAP nanoparticles act as fillers for the small cavities present within the degraded bone; the subsequent application of the larger Ca(OH)_2_ nanoparticles dispersed in 2-propanol followed by an aqueous solution of DAP, leads to the *in-situ* formation of a continuous matrix of HAP and perhaps CaCO_3_ that, during crystallisation, both act as binders in a sort of mortar, where the inert phase is now consisted of the previously applied HAP nanoparticles.

### Palaeogenetic analysis

To investigate the impact of the consolidation protocol on the yield of human aDNA, the results of the palaeogenetic analysis from treated and untreated fragments were compared. The reconstruction of the complete mitochondrial genome from both T and NT fragments was successful only for two out of four individuals (Muŝov66 and Muŝov71) due to lower DNA preservation in the remaining samples (Muŝov65 and Muŝov73b) (Table 2, Table SI4). Indeed, we found that Muŝov65 exhibits a very low preservation of endogenous DNA and returned very low-quality data in both T and NT fragments that precludes meaningful comparison. With the exception of Muŝov66, the percentage of endogenous DNA slightly decreased in the consolidated fragments with respect to the corresponding untreated ones. Muŝov73b showed the largest decrease, with an amount of endogenous DNA of 5.84% in the untreated sample and of 1.20% in the treated one. The same trend was also detected when considering the mean coverage, which corresponds to the average number of unique DNA sequences covering each position of the reference genome. In Muŝov66 the mean coverage was slightly higher in the consolidated fragment (33.83X) than in the untreated one (24.95X), while the depth of coverage decreased in the consolidated fragments of the other samples with Muŝov73b showing the most detrimental impact (from 6.08X to 0.43X in consolidated and untreated fragments respectively).

**Table 2:** Results of the analysis performed on the mitochondrial DNA recovered from the untreated (NT) and treated (T) bone samples coming from the Longobard site of Muŝov.

Sample Name	Endogenous DNA (%)	Mean Coverage (X)	Coverage ≥ 1X (%)	Coverage ≥ 5X (%)	Missing position in the consesnsus sequence	DMG 1st base 3'	DMG 1st base 5'	Average fragment length (bp)	Proportion of authentic reads (%)—ContamMix	mtDNA haplogroup	# of variants
Muŝov65-NT	1.49	1.21	65.08	2	5887	0.32	0.38	49.08	99.43	T2	17
Muŝov65-T	0.66	0.42	31.35	0	11.461	0.43	0.25	45.56	96.69	–	1
Muŝov66-NT	16.93	24.95	100	99.89	2	0.32	0.31	50.97	98.83	T2e	36
Muŝov66-T	22.56	33.83	100	99.91	1	0.29	0.32	51.73	99.97	T2e	35
Muŝov71-NT	5.13	11.21	100	95.30	3	0.34	0.35	49.45	99.29	H1c1	11
Muŝov71-T	4.54	8.32	99.96	87.08	11	0.33	0.35	51.55	99.69	H1c1	12
Muŝov73b-NT	5.84	6.08	99.50	67.31	103	0.38	0.36	49.79	99.81	H1c1	14
Muŝov73b-T	1.20	0.43	34.79	0	10.850	0.36	0.29	46.20	98.40	–	3

These observations were also confirmed by the onefold and fivefold mitogenome coverage parameters, representing the percentage of mitochondrial genome positions covered by at least 1 and 5 reads, respectively. Over 99% of the mitochondrial genome of both Muŝov66-T and Muŝov66-NT was covered at least fivefold. At the same coverage good percentages were also observed for Muŝov71-T (95.30%), Muŝov71-NT (87.08%) and Muŝov73b-NT (67.31%), while no data was available for the remaining analysed samples. On the contrary, the low percentages of mitochondrial genome covered at least onefold in Muŝov73b-T (34,79%), as well as in Muŝov65-NT (65,08%) and Muŝov65-T (31,35%), indicated that a large portion of the mitochondrial genome is missing. Starting from the reconstructed consensus sequence, it was possible to determine the mitochondrial haplogroup from both T and NT fragments only in the case of Muŝov66 and Muŝov71. For each individual both fragments returned exactly the same mtDNA haplogroup and the same variants with respect to the reference sequence (rCRS) except a single position in a homopolymeric C-stretch, proving that the consolidation procedure did not affect the reliability of the results in the samples exhibiting a degree of DNA preservation suitable for palaeogenetic analysis. However, the accurate determination of the mitochondrial haplogroup from the consolidated samples Muŝov65-T and Muŝov73b-T was not possible due to the high number of missing positions in the consensus sequence (more than 10.000 missing positions out of a total mtDNA length of 16.569 bp). Instead, haplogroup identification was successfully performed from the untreated fragments despite the presence of several missing positions in the consensus sequence of Muŝov65-NT. The full analyses repeated after performing the down sampling on the two best preserved samples Muŝov66 and Muŝov71 confirm the observations reported so far, excluding any possible bias in the interpretation of the results associated with the different number of raw reads generated by sequencing (Table SI5).

For what concerns the assessment of the damage patterns, no relevant variation was detected between treated and untreated samples in terms of deamination rates (namely, proportion of damaged cytosine at both 5’ and 3’ ends of DNA molecules) and average fragment length, which were both in line with values expected for degraded ancient DNA samples. The results of the ContamMix test showed very similar proportion of authentic reads in both consolidated and untreated samples, demonstrating that no significant contamination with modern DNA was introduced by applying the treatment. The slight decrease in the percentage of authentic reads observed forMuŝov65-T and of Muŝov73b-T was likely to derive from the low amount of mtDNA retrieved from these samples, which surely represents a limiting factor for the analysis. In fact, the software was not able for these two samples, as well as for Muŝov65-NT, to calculate a precise estimate of the percentage of authentic reads, as can be noticed considering the wide distribution obtained in a 95% confidence interval (Table SI4).

Despite the high variability within the samples, the overall result may suggest that consolidation protocol does not significantly affect the quality of the genetic data in adequately well-preserved samples, where the same consensus sequences was obtained from both treated and untreated fragments. On the contrary, the consolidation treatment seems to potentially affect the yield of the palaeogenetic analysis when applied on specimens with particularly scarce DNA preservation. Additional bones with different conservation conditions need to be tested to confirm this hypothesis.

### Radiocarbon analysis

Table 3 shows the AMS radiocarbon results: for each of the bones, the radiocarbon concentrations measured in the untreated (NT) and in the HAP treated (T) fractions are compared. In the case of the sample from *Porticus Octaviae* (P.O.us898), the radiocarbon concentration measured in a Paraloid-treated sample is also reported.

**Table 3:** ^14^C-AMS results: radiocarbon concentrations measured in untreated (NT) and HAP treated (T) fractions. In the case of the sample from Porticus Octaviae (P.O.us898), the radiocarbon concentration measured in a Paraloid-treated sample is also reported.

	NT	T	Paraloid-T
	^14^C conc. (pMC)	^14^C conc. (pMC)	^14^C conc. (pMC)
Muŝov65	83.74 ± 0.69	82.71 ± 0.44	
Muŝov66	83.03 ± 0.35	82.81 ± 0.47	
Muŝov73b	82.75 ± 0.34	82.58 ± 0.51	
P.O.us898	88.25 ± 0.40	88.42 ± 0.22	82.95 ± 0.54

The comparison of the NT data with the T data showed that no contamination due to the HAP treatment was detected. Indeed, both T and NT fractions, as well as P.O.us898 Paraloid-treated sample, were prepared for the ^14^C measurement following the aforementioned procedure based on HCl and NaOH solutions, that are meant to achieve the complete demineralisation of the bone and its purification from natural humic substances but are likely to have a poor effect on anthropogenic contaminations. The consolidation by HAP treatment thus appeared as a safe operation, which can assure good radiocarbon results also in those cases when no information about previous restorations has been given to the dating laboratory. In fact, the applied preparation procedure is basically the standard approach for any bone sample collected from an archaeological context before radiocarbon dating.

In the case of the P.O.us898 Paraloid-treated sample, a lower concentration than the one observed in the corresponding NT fraction was measured. This was somehow expected, since Paraloid is typically synthesised from low-^14^C materials (i.e., materials rich in fossil carbon whose radiocarbon concentration is well below the sensitivity limits of the measurement technique), and it cannot be removed by just applying the acidic and basic solutions used in the preparation procedure described above. However, some preparation strategies exploiting organic volatile solvents can be used to get rid of such a contamination, even though these procedures are more time consuming and challenging.

## Conclusions

In this study, we set up an innovative and easily applicable protocol for the consolidation of ancient bones based on the use of inorganic nanostructured materials with high physico-chemical compatibility with the bone matrix. The goal was to recover mechanical properties lost due the damages induced by interactions with the external that cause an increase of the porosity and a consequent decrease of density and mechanical resistance. We also aimed at defining a consolidation protocol that did not compromise the results of molecular analyses, such as ^14^C radiocarbon dating and palaeogenetic analysis, that can be carried out on archaeological and historical bone remains.

Our results suggest the availability of an easy and low-cost method that will be implemented for production on a large scale in further studies, also in the view of evaluating the use of these products for other applications (i.e. in the biomedical field).

From the physico-chemical standpoint, several analytic tests proved the effectiveness of the proposed consolidation protocol. SEM data indicated an increase of the homogeneity of the structure of the bone induced by the consolidation treatment. These data were confirmed by microtomography, which showed an increase of the density and a decrease of the total porosity of the treated bones, not only on the surface of the samples, but also in the bulk of the porous network of the bone, indicating a deep penetration of the treatment. In addition, the increment of the Vicker micro-hardness also confirmed the improvement of the physical–mechanical properties of the consolidated bones. All the results evidenced that the four main open points derived from a previous work^28^ were cleared and the new methodology revealed itself original, novel, and improved.

One of the key features of this work is the evaluation of the impact of the proposed protocol on the results of two analyses typically performed on ancient bone remains, namely palaeogenetic analysis and radiocarbon dating. In agreement with the results of a previous study based on a similar approach^28^, mitogenome reconstruction from both untreated and consolidated bone fragments have shown that the consolidation protocol does not significantly affect the quality of the genetic results in bones with adequate DNA preservation. However, we noticed that the treatment might have a negative impact on the endogenous DNA yield and on subsequent genetic results when applied on samples with a particularly poor amount of preserved genetic material (such as Muŝov65 and Muŝov73b). Although additional tests on a larger set of bones with different conservation conditions need to be carried out to better address this issue, we caution to apply the consolidation protocol here developed on bone fragments that could represent unique remains. It is worthwhile to specify that, at the state of the art, palaeogenetic analyses are preferably performed on the petrous part of the temporal bones rather than long bones because endogenous DNA is known to be much better preserved and protected from exogenous contamination in that skeletal element^59^. Therefore, the evidence of a potential negative influence of the consolidant on extremely degraded long bones (that could benefit from this treatment) does not exclude the possibility of carrying on palaeogenetic analysis on other skeletal districts, such as the petrous bone, when available for study. The evaluation of the impact of consolidation on radiocarbon dating represents one of the most important novelty of this study. The results have shown that no contamination due to the HAP treatment is detected, thus avoiding the need for time-consuming decontamination protocols during sample preparation.

In conclusion, the protocol developed in this study offers a more compatible alternative to traditional consolidation based on organic polymers and to inorganic treatments reported in previous studies. Even if additional studies will be necessary to improve the applicative procedures, the treatment is easily-applicable and compatible with different molecular analyses. Such exhaustive and informative evaluation of the performance and the effects of the proposed consolidation protocol was achieved integrating expertises from different scientific fields, showing the benefit of a multidisciplinary approach to face problems and individuate effective solutions in the field of cultural heritage conservation.

## Supplementary Information

Supplementary Information.
