# Supplementary material for: Performance of innovative nanomaterials for bone remains consolidation and effect on 14C dating and on palaeogenetic analysis
Source: Sci Rep. 2022 Apr 28;12:6975. doi: 10.1038/s41598-022-10798-5 (PMC9050738; doi:10.1038/s41598-022-10798-5)
Supplement: Supplementary file 1 — Supplementary Information. [file 41598_2022_10798_MOESM1_ESM.docx]

**SUPPORTING INFORMATION**

Performance of innovative nanomaterials for bone remains consolidation and effect on ^14^C dating and on palaeogenetic analysis

Francesca Porpora^1^, Valentina Zaro^2^, Lucia Liccioli^3^, Arianna Meoli^1^, Giulia Marradi^1^, Serena Barone^3^, Alessandra Modi^2^, Stefania Vai^2^, Luigi Dei^1^, David Caramelli^2^, Mariaelena Fedi^3^, Martina Lari^2^, Emiliano Carretti^1^

^1^*Department of Chemistry “Ugo Schiff” and CSGI, University of Florence, Via della Lastruccia 3, 50019, Sesto Fiorentino, FI, Italy*

^2^*University of Florence, Department of Biology, via del Proconsolo 12, 50122 Firenze, Italy*

^3^*INFN (Istituto Nazionale di Fisica Nucleare) Sezione di Firenze, Via Sansone 1, 50019, Sesto Fiorentino, FI, Italy*

**1. Skeletal materials**

**Table SI1.** Human bone remains selected for the evaluation of the consolidation treatment.

| **Sample code** | **Archeological site** | **Bone district** | **Analyses** | | |
| --- | --- | --- | --- | --- | --- |
|  |  |  | **Physico-chemical** | **Paleogenetical** | **Radiocarbon dating** |
| Mušov65 | Mušov (Czech Republic) | Femur |  | X | X |
| Mušov66 | Mušov (Czech Republic) | Femur | X | X | X |
| Mušov71 | Mušov (Czech Republic) | Femur |  | X |  |
| Mušov73b | Mušov (Czech Republic) | Femur |  | X | X |
| P.O.us898 | Porticus Octaviae (Italy)1 | Humerus |  |  | X |
| **2. Characterization of products** | | | | | |

The chemical nature of the synthesized materials has been investigated also through FTIR that has been carried out on all the powders in transmittance mode in the range 4000-400 cm^-1^ (Fig. SI1A). In particular, a weak peak associated with the P-O symmetric stretching is visible at ~ 996 cm^-1^ and 982 cm^-1^ for samples obtained from syntheses 1 and 2 respectively, and at 962 cm^-1^ for syntheses 3 and 4. The position of this peak allows discriminating between the phases formed through the different syntheses procedures. In fact, as reported in literature, while the peak at 962 cm^-1^ is characteristic of hydroxyapatite, the band at ~ 995 cm^-1^ and at ~985 cm^-1^ are typical of monetite and brushite respectively [1] (Fig. SI1B), preliminarily indicating that only through syntheses 3 and 4 hydroxyapatite is formed selectively. All the spectra show the typical bands of calcium phosphate, that are listed in Table SI1 with the corresponding attributions [2, 3, 4, 5].

| 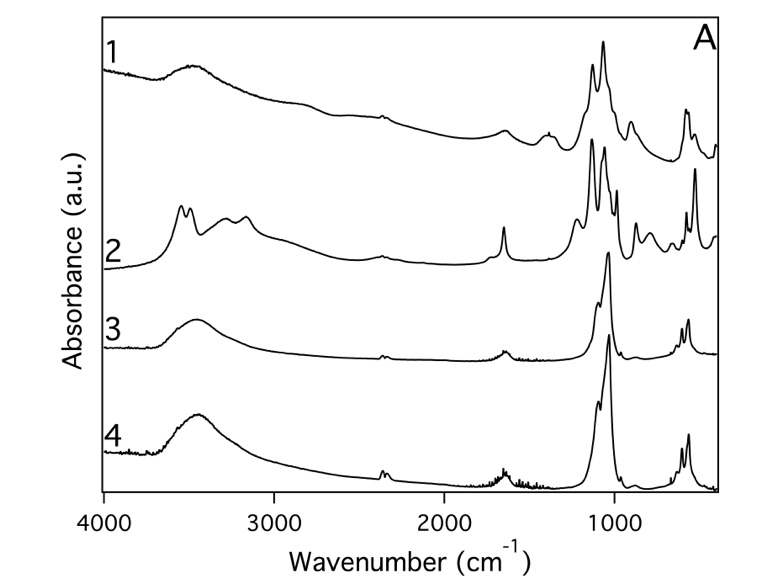 | 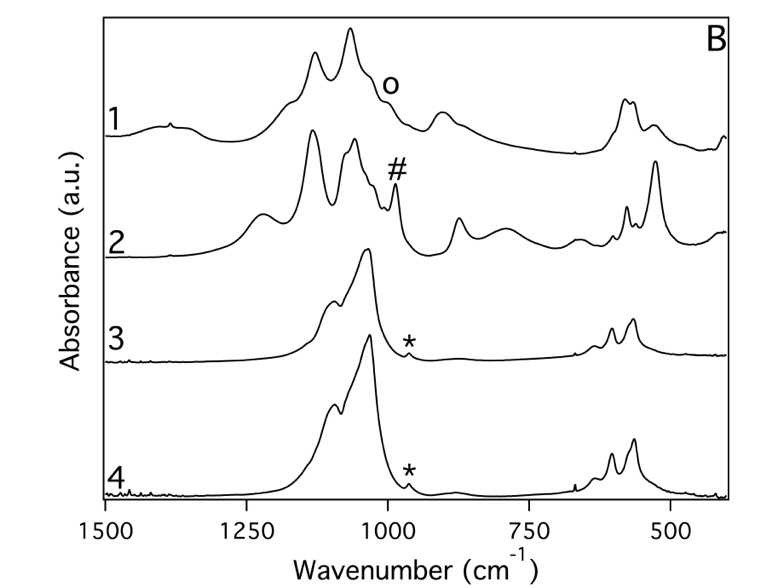 |
| --- | --- |

**Figure SI1.** (A) FTIR spectra (4000-400 cm^-1^) of calcium phosphates obtained from syntheses indicated in Table 1. (B) FTIR spectra (1500-400 cm^-1^). The symbols indicate the peak associated with the P-O symmetric stretching at 960 and 964 cm^-1^ (*), which is typical for hydroxyapatite; when the peak is shifted to 996 cm^-1^ (°) and 982 cm^-1^ (#), it points to the presence of monetite and brushite, respectively.

**Table SI2.** Wavenumbers for functional groups of the particles obtained from syntheses 1-4 (cm^-1^).

| Samples | | | | Assignment |
| --- | --- | --- | --- | --- |
| 1 | 2 | 3 | 4 |  |
| Wavenumber (cm^-1^) | | | |  |
|  | 3542 |  |  | ν OH |
|  | 3491 |  |  | ν OH |
| 3460 |  | 3566 | 3566 | ν OH |
|  | 3285 |  |  | ν OH |
| 3209 |  |  |  | ν OH |
|  | 3162 |  |  | ν OH |
| 2803 |  |  |  | ν OH |
|  | 1724 |  |  | δ OH^-^ |
| 1641 | 1650 | 1641 | 1646 | δ OH^-^ |
| 1384 |  |  | 1456 | ν_as_ CO_3_^2-^/  δ HPO_4_^2-^ |
| 1185 | 1222 |  |  | δ HPO_4_^2-^ |
| 1128 | 1131 |  |  | ν HPO_4_^2-^ |
| 1064 | 1060 | 1097 | 1093 | ν_as_ PO_4_^3-^ |
| 1031 | 1027 | 1037 | 1031 | ν_as_ PO_4_^3-^ |
|  | 1004 |  |  | ν PO_4_^3-^ |
| 996 | 982 | 960 | 964 | ν PO_4_^3-^ |
| 900 | 872 |  |  | δ CO_3_^2-^/  ν HPO_4_^2-^ |
|  | 789 |  |  | OH^-^ (libration) |
|  | 660 | 660 | 668 | CO_2_ |
|  |  | 636 | 633 | OH^-^ (libration) |
|  | 602 | 602 | 602 | δ PO_4_^3-^ |
| 580 | 576 |  |  | δ PO_4_^3-^ |
| 564 | 564 | 564 | 564 | δ PO_4_^3-^ |
| 527 | 523 |  |  | δ PO_4_^3-^ |
| 404 | 408 |  |  | δ PO_4_^3-^ |


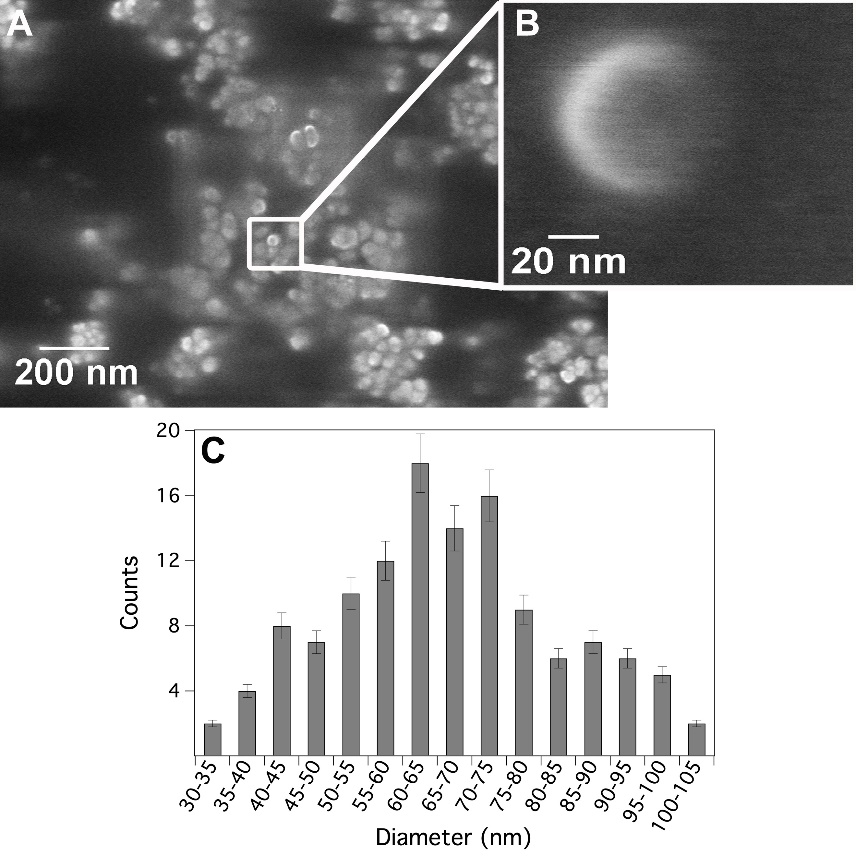


**Figure SI2.** SEM micrographs of HAP nanoparticles obtained from synthesis 3 collected at different magnifications: 10^5^ X (A) and 10^6^ X (B); (C) size distributions of HAP nanoparticles from synthesis 3 obtained by the SEM micrographs and elaborated through ImageJ.


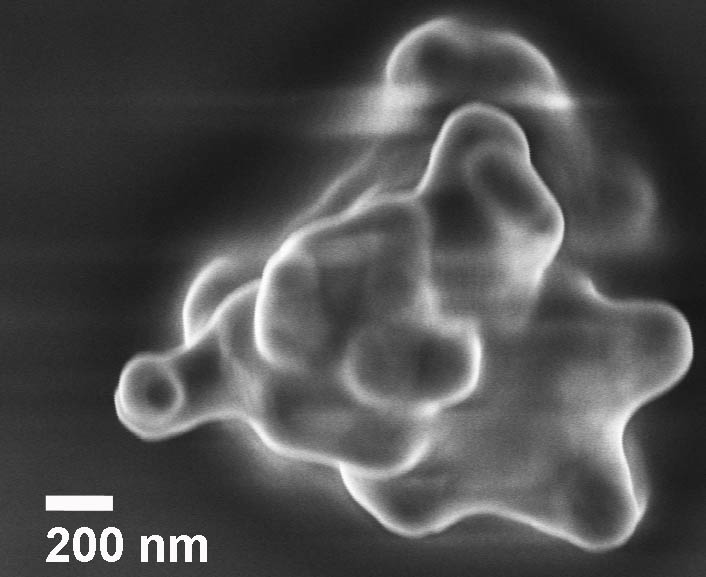


**Figure SI3.** SEM micrograph registered at 100 kx of magnitude of an agglomerate of HAP particles obtained under the same conditions of synthesis 4 (Table 1) but in the absence of the ultrasonic bath.

The stability of the dispersions of HAP nanoparticles in 2-propanol was evaluated through turbidimetry measurements (Figure SI4). Comparing them to dispersions in a different solvent like water. This is a key point because the settling process is strictly related to the rate of aggregation of the nanocrystals: as faster is the sedimentation of the nanoparticles, as higher is the aggregation degree that inhibits their penetration into the porous matrix of the bone. From Figure SI4A, it is evident that HAP nanoparticles dispersed in 2-propanol result stable up to 2500 s, while in water they sediment after few minutes.

From a qualitative observation of the two dispersions in water and in 2-propanol (Fig. SI4B), the one in 2-propanol seems to be still stable even after five hours indicating a low tendency to the aggregation of the HAP nanoparticles dispersed in this solvent. Because of similar polarity, this behavior is probably comparable to that shown by analogous dispersions of Ca(OH)_2_ nanoparticles [6]. The water molecules interact with the phosphate groups of the HAP nanoparticles and intercalate between them through the formation of hydrogen bonds, promoting their aggregation. On the contrary, in 2-propanol, hydroxyl groups are absorbed on the surface of the nanoparticles and the aggregation is inhibited thanks to the low interaction between the short aliphatic chains.

| 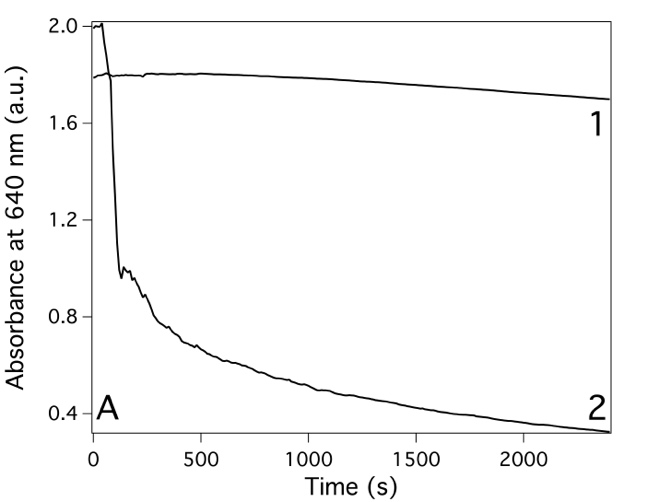 |
| --- |
| 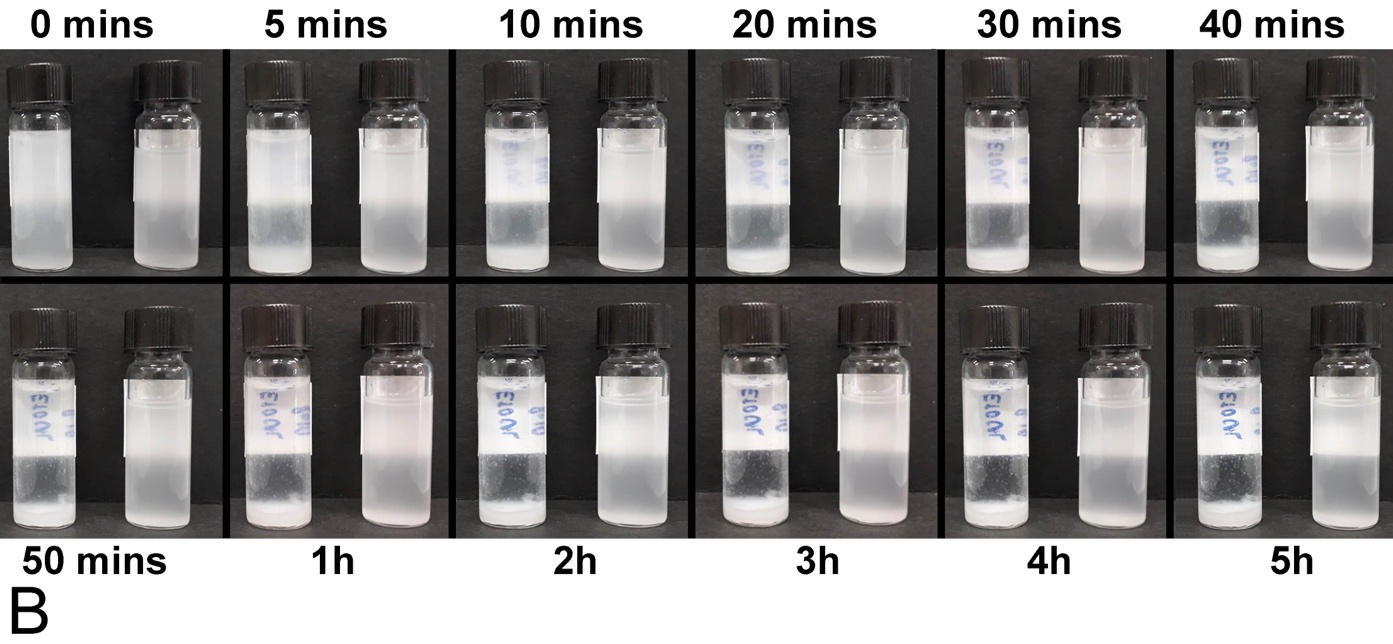 |

**Figure SI4**. (A) Absorbance at 640 nm as a function of time for HAP nanoparticles from synthesis 4 dispersed in 2-propanol (1) and water (2); (B) Stability of the HAP nanoparticles from synthesis 4 dispersed in water (left) and 2-propanol (right) between 0 and 5 hours. While nanoparticles dispersed in water start to sediment after few minutes, those dispersed in 2-propanol appear still stable even after five hours.

**3. Evaluation of the impact of the consolidation treatment on the physico-chemical and mechanical properties of the bone**

**
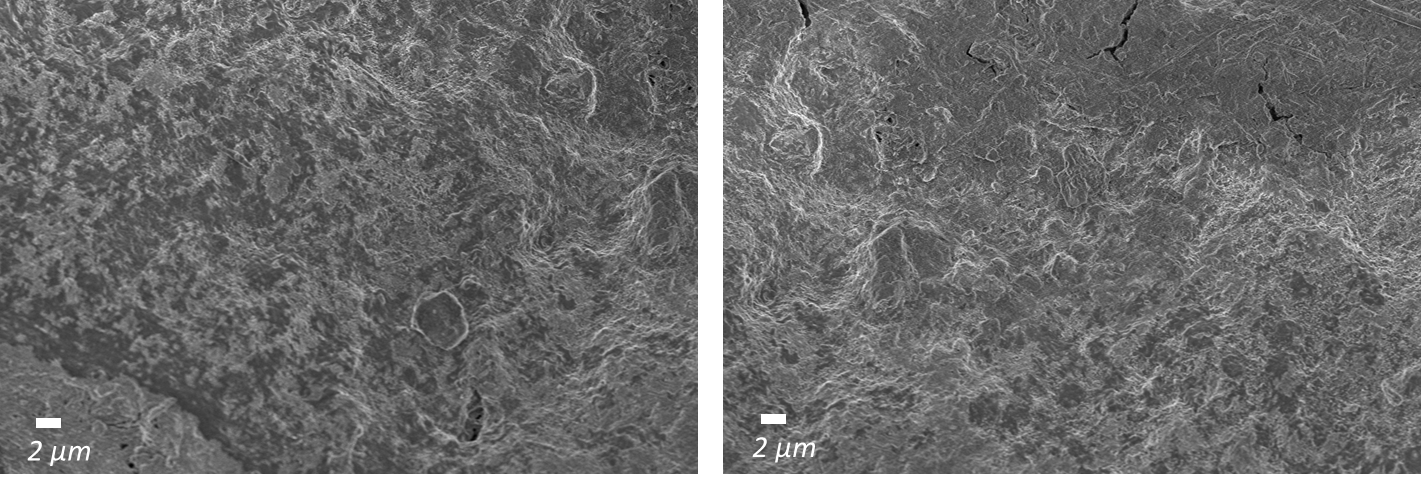
**

**Figure SI5.** SEM micrographs registered at 5 kx of magnitude of the sample from Muŝov66 acquired after the treatment.

**
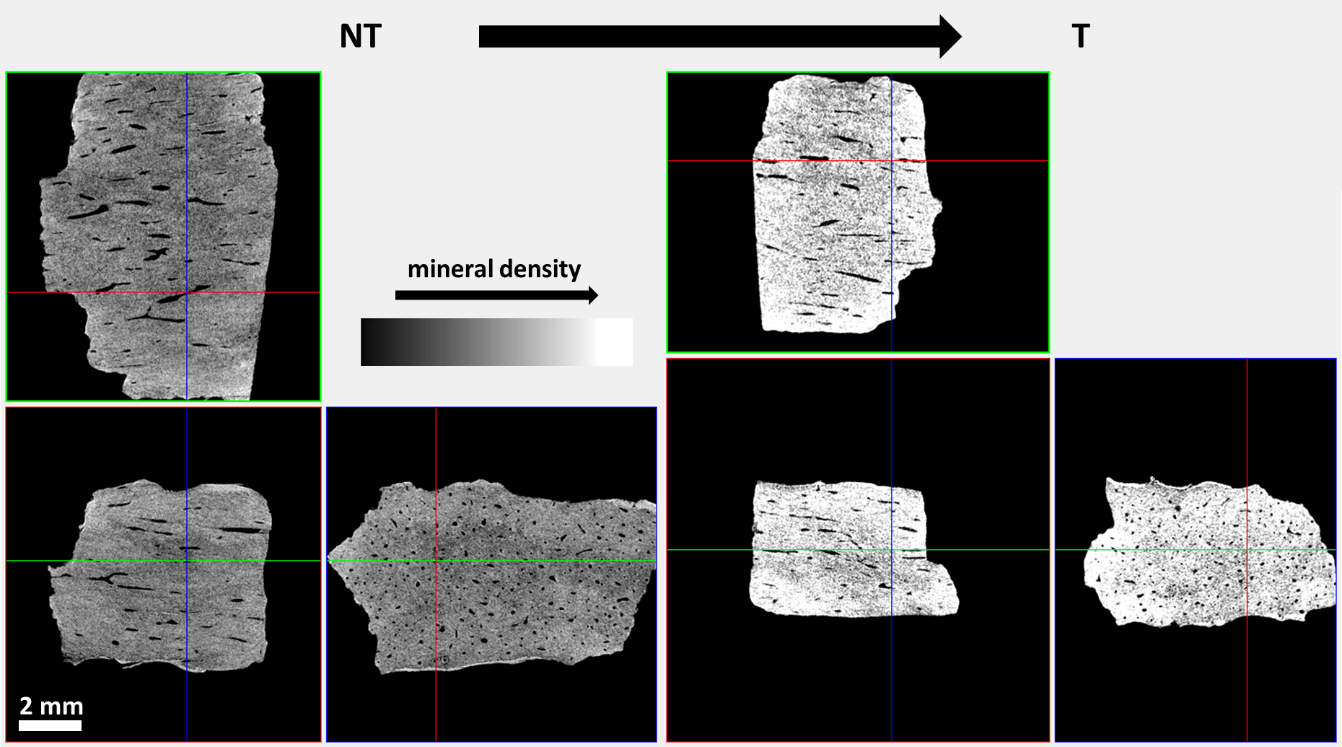
**

**Figure SI6.** Sections along three axes of a 3D reconstructions of a bone fragment from the archaeological site of Muŝov (Mušov 66) before (left) and after (right) the consolidation treatment: it is possible to appreciate an increase in the mineral density in both the surface and the bulk of the sample.

**Table SI3.** Porosity data obtained from microtomography analysis performed on the untreated and the treated samples

|  | Sample name | |
| --- | --- | --- |
|  | NT | T |
| Total volume of pores (mm^3^) | 4.28 | 1.46 |
| Total Porosity (%) | 3.84 | 2.25 |
| Volume of closed pores (mm^3^) | 0.40 | 0.61 |
| Closed Porosity (%) | 0.38 | 0.96 |
| Volume of open pores (mm^3^) | 3.87 | 0.85 |
| Open Porosity (%) | 3.48 | 1.31 |

The nitrogen adsorption-desorption isotherms (Fig. SI7A), recorded from bone fragments of 1-2 millimeters, give information about the pore size distribution in the range of 0.4 to 200 nm in diameter. According to IUPAC classification [7], all the bone samples exhibit a type IV(a) isotherm, characteristic of mesoporous materials with pores ranging from 2 to 50 nm in pore size and a type H3 hysteresis loop, given by non-rigid aggregates of plate-like particles. This kind of loop is typical of pores with irregular size and shape, suggesting the presence of slit-like pores [7, 8].

By comparing the profiles of the isotherms acquired from NT and T samples, the volume of absorbed gas is lower in the consolidated sample:

- at low partial pressures (up to 0.2), indicating the filling of the micropores;
- after the inflection point, suggesting the reduction of available area for multilayer adsorption due to the formation of a homogeneous coating.

In Fig. SI7B, the graph shows the size distribution of pores obtained from the desorption isotherm before and after the treatment: although the model applied [9] considers the presence of cylindrical pore, which is not our case, it is possible to appreciate a decrease of the differential volume for pores with diameter up to 50 nm. Moreover, Figure SI7B indicates that the trend of the differential pore volume as a function of the pore diameter doesn’t change meaningfully after the consolidation treatment, suggesting a homogeneous distribution of the consolidant inside of the bone matrix.

In particular, on the basis of what we reported in a previous paper [8], the decrease of the differential pore volume in the range of micro- and mesopores, can be mainly attributed to the precipitation of phosphate salts deriving from the DAP solution in the narrowest pores.

| 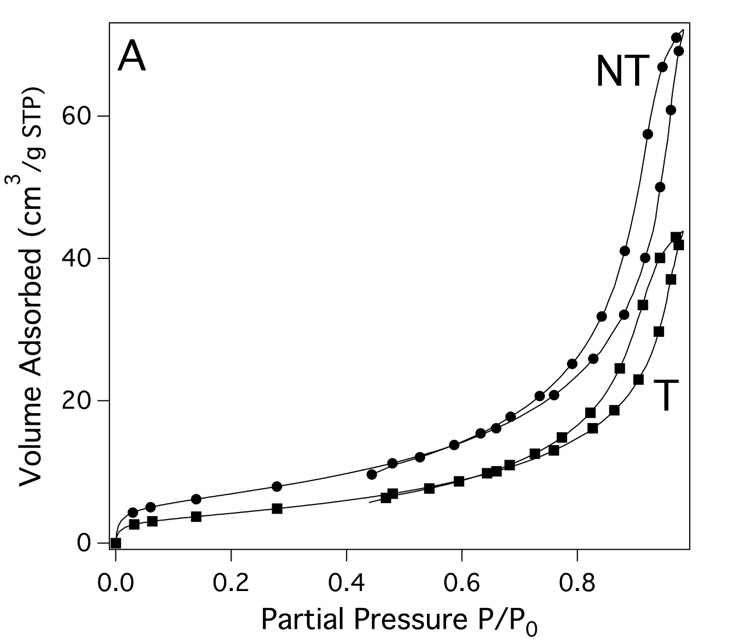 | 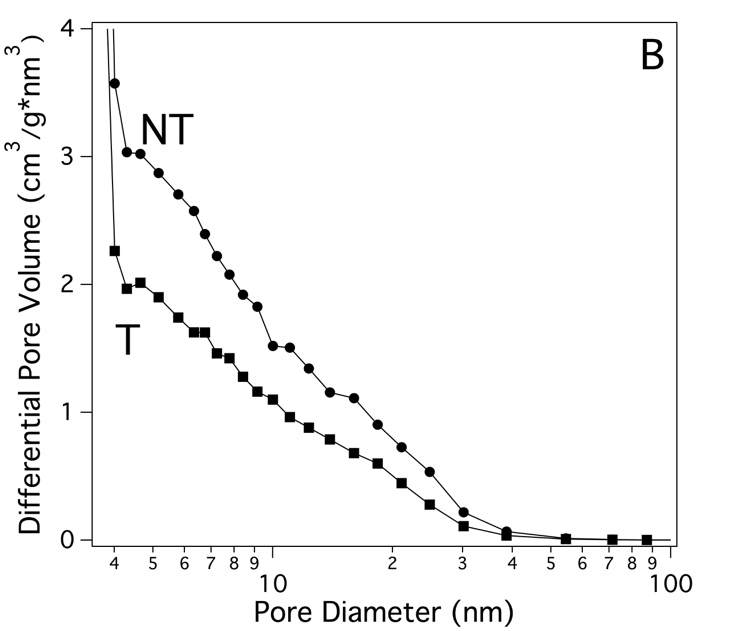 |
| --- | --- |

**Figure SI7.** (A) BET adsorption-desorption isotherms for bone samples from Muŝov obtained before (NT) and after (T) the consolidation treatment; (B) Pore diameter distribution plots for bone samples from Muŝov before (NT) and after (T) the consolidation treatment.

**4. Paleogenetic analysis**

**Table SI4.** Full sequencing and bioinformatic results of the mitochondrial genomes obtained from the untreated (NT) and treated (T) bone samples from Muŝov.

| **Sample Name** | **Raw reads prior Clip&Merge** | **Merged reads** | **% Merged Reads** | **Mapped reads prior duplicates removal** | **Mapped Reads after  duplicates removal** | | **% Endogenous DNA** | **Cluster factor** |
| --- | --- | --- | --- | --- | --- | --- | --- | --- |
| Musov65-NT | 1889070 | 562097 | 50,67 | 8391 | 410 | 1,49 | | 20,47 |
| Musov65-T | 1482784 | 422262 | 47,84 | 2793 | 154 | 0,66 | | 18,14 |
| Musov66-NT | 2766938 | 993235 | 67,71 | 168192 | 8111 | 16,93 | | 20,74 |
| Musov66-T | 3466068 | 1277411 | 69,95 | 288125 | 10835 | 22,56 | | 26,59 |
| Musov71-NT | 2863170 | 1059589 | 70,11 | 54365 | 3757 | 5,13 | | 14,47 |
| Musov71-T | 1951798 | 633975 | 56,42 | 28775 | 2675 | 4,54 | | 10,76 |
| Musov73b-NT | 2040470 | 619621 | 52,38 | 36167 | 2025 | 5,84 | | 17,86 |
| Musov73b-T | 762148 | 203254 | 43,54 | 2447 | 153 | 1,20 | | 15,99 |

| **Sample Name** | **Mean Coverage** | **Standard deviation coverage** | **Coverage ≥ 1X (%)** | **Coverage ≥ 2X (%)** | **Coverage ≥ 3X (%)** | **Coverage ≥ 4X (%)** | **Coverage ≥ 5X (%)** |
| --- | --- | --- | --- | --- | --- | --- | --- |
| Muŝov65-NT | 1,21 | 1,25 | 65,08 | 33,99 | 14,04 | 5,54 | 2 |
| Muŝov65-T | 0,42 | 0,73 | 31,35 | 8,49 | 1,91 | 0,59 | 0 |
| Muŝov66-NT | 24,95 | 8,72 | 100 | 100 | 100 | 99,94 | 99,89 |
| Muŝov66-T | 33,83 | 10,62 | 100 | 99,99 | 99,99 | 99,99 | 99,91 |
| Muŝov71-NT | 11,21 | 4,27 | 100 | 99,80 | 99,31 | 98,09 | 95,30 |
| Muŝov71-T | 8,32 | 3,49 | 99,96 | 99,65 | 98,47 | 94,39 | 87,08 |
| Muŝov73b-NT | 6,08 | 2,89 | 99,50 | 96,68 | 90,13 | 79,87 | 67,31 |
| Muŝov73b-T | 0,43 | 0,65 | 34,79 | 6,71 | 1,04 | 0,11 | 0 |

| **Sample Name** | **DMG 1st  base 3'** | **DMG 1st  base 5'** | **Average fragment  length (bp)** | **Missing position in the consensus sequence** | **Proportion of authentic reads (%) - ContamMix** | **Proportion of authentic reads (%) CI (2.5%-97.5%) - ContamMix** | | **mtDNA  haplogroup** |
| --- | --- | --- | --- | --- | --- | --- | --- | --- |
| Muŝov65-NT | 0,32 | 0,38 | 49,08 | 5887 | 99,43 | 87,46-99,91 | T2 | |
| Muŝov65-T | 0,43 | 0,25 | 45,56 | 11461 | 96,69 | 42,65-99,39 | - | |
| Muŝov66-NT | 0,32 | 0,31 | 50,97 | 2 | 98,83 | 96,24-99,79 | T2e | |
| Muŝov66-T | 0,29 | 0,32 | 51,73 | 1 | 99,97 | 99,20-99,99 | T2e | |
| Muŝov71-NT | 0,34 | 0,35 | 49,45 | 3 | 99,29 | 94,18-99,90 | H1c1 | |
| Muŝov71-T | 0,33 | 0,35 | 51,55 | 11 | 99,69 | 95,06-99,94 | H1c1 | |
| Muŝov73b-NT | 0,38 | 0,36 | 49,79 | 103 | 99,81 | 95,61-99,97 | H1c1 | |
| Muŝov73b-T | 0,36 | 0,29 | 46,20 | 10850 | 98,40 | 69,06-99,71 | - | |

| **Sample Name** | **Reads prior mapping** | **Mapped reads prior  duplicates removal** | **Mapped reads after  duplicates removal** | **% Endogenous**  **DNA** | | **Mean**  **Coverage** | **Coverage ≥ 1X**  **(%)** | **Coverage ≥ 5X**  **(%)** |
| --- | --- | --- | --- | --- | --- | --- | --- | --- |
| Muŝov66-NT | 993235 | 168192 | 8111 | 16,93 | 24,9508 | | 100 | 99,89 |
| Muŝov66-T | 993235 | 224249 | 10709 | 22,58 | 33,4548 | | 100 | 99,82 |
| Muŝov71-NT | 633975 | 32471 | 3606 | 5,12 | 10,7963 | | 100 | 94,65 |
| Muŝov71-T | 633975 | 28775 | 2675 | 4,54 | 8,3226 | | 99,96 | 87,08 |
|  |  |  |  |  |  | |  |  |

**Table SI5.** Results obtained from the EAGER pipeline after having downsampled the merged data of Muŝov66-T and Muŝov71-NT to a number of 993235 and 633975 reads respectively.

References

| [1] | C. Drouet, «Apatite Formation: Why It May Not Work as Planned, and How to Conclusively Identify Apatite Compounds,» *BioMed Research International,* vol. 2013, pp. 1-12, 2013. |
| --- | --- |
| [2] | L. Berzina-Cimdina e N. Borodajenko, «Research of Calcium Phosphate Using Fourier Transform Infrared Spectroscopy,» in *Infrared Spectroscopy - Material Science, Engineering and Technology*, InTech, 2012. |
| [3] | S. Mansour, S. El-Dek, M. Ahmed, S. Abd-Elwahab e M. Ahmed, «Effect of preparation conditions on the nanostructure of hydroxyapatite and brushite phases,» *Applied Nanoscience (Switzerland),* vol. 6, n. 7, pp. 991-1000, 2016. |
| [4] | N. Nga, N. Chau e P. Viet, «Facile synthesis of hydroxyapatite nanoparticles mimicking biological apatite from eggshells for bone-tissue engineering,» *Colloids and Surfaces B: Biointerfaces,* vol. 172, pp. 769-778, 2018. |
| [5] | B. Cengiz, Y. Gokce, N. Yildiz, Z. Aktas e A. Calimli, «Synthesis and characterization of hydroxyapatite nanoparticles,» *Colloids and Surfaces A: Physicochemical and Engineering Aspects,* n. 1-3, pp. 29-33, 2008. |
| [6] | E. Fratini, G. Page, R. Giorgi, H. Colfen, P. Baglioni e B. e. Z. T. Demé, «Competitive Surface Adsorption of Solvent Molecules and Compactness of Agglomeration in Calcium Hydroxide Nanoparticles,» *Langmuir,* vol. 23, n. 5, p. 2330–2338, 2007. |
| [7] | M. Thommes, K. Kaneko, A. Neimark, J. Olivier, F. Rodriguez-Reinoso, J. Rouquerol e K. Sing, «Physisorption of gases, with special reference to the evaluation of surface area and pore size distribution (IUPAC Technical Report),» *Pure and Applied Chemistry,* 2015. |
| [8] | A. Salvatore, S. Vai, S. Caporali, D. Caramelli, M. Lari e E. Carretti, «Evaluation of Diammonium hydrogen phosphate and Ca(OH)2 nanoparticles for consolidation of ancient bones,» *Journal of Cultural Heritage,* vol. 41, n. 2019, pp. 1-12. |
| [9] | P. Ravikovitch e A. Neimark, «Calculations of pore size distributions in nanoporous materials from adsorption and desorption isotherms,» *Studies in Surface Science and Catalysis,* vol. 129, pp. 597-606, 2000. |
